# Supplementary material for: Vinasse application and cessation of burning in sugarcane management can have positive impact on soil carbon stocks
Source: PeerJ. 2018 Aug 7;6:e5398. doi: 10.7717/peerj.5398 (PMC6086084; doi:10.7717/peerj.5398)
Supplement: Supplemental Information 1 — All supplemental information attached to the docx document [file peerj-06-5398-s001.docx]

**SUPPORTING INFORMATION**

**Table S1:** Soil textural properties and bulk density in the no vinasse (NV) and vinasse (V) and burned (B) and unburned (UB) systems up to 1 metre depth. Standard error for soil textural n = 3, while for bulk density and carbon content n = 12 for 0-10,10-20 and 20-30 cm and n = 6 for the remaining layers.

| **Reference area** | | **Depth** | **Sand** | **Silt** | **Clay** | **Bulk density** | **C content** |
| --- | --- | --- | --- | --- | --- | --- | --- |
|  |  | cm | g kg^-1^ | | | g cm^-3^ | g kg^-1^ |
| No vinasse application  (NV) | | 0-10 | 98.7±1.1 | 205.3±2.9 | 696.7±4.3 | 1.1±0.01 | 18.77±0.1 |
|  |  | 10-20 | 91.3±1.1 | 208.7±5.4 | 700.0±4.3 | 1.08±0.01 | 16.93±0.1 |
|  |  | 20-30 | 82.7±1.5 | 166.3±0.4 | 751.0±1.9 | 1.12±0.00 | 13.84±0.1 |
|  |  | 30-40 | 88.7±0.4 | 203.0±1.9 | 708.3±1.7 | 1.14±0.02 | 10.35±0.6 |
|  |  | 40-50 | 100.3±1.7 | 278.3±1.1 | 621.3±1.1 | 1.04±0.04 | 11.77±1.1 |
|  |  | 50-60 | 92.0±1.9 | 203.7±0.4 | 704.3±2.1 | 1.04±0.02 | 10.02±0.5 |
|  |  | 60-70 | 119.0±0.7 | 194.7±1.1 | 686.3±0.4 | 0.99±0.02 | 9.32±0.3 |
|  |  | 70-80 | 116.3±0.1 | 204.7±0.1 | 679.0±0.0 | 0.95±0.02 | 9.88±0.3 |
|  |  | 80-90 | 111.7±2.1 | 186.0±1.9 | 702.3±4.0 | 0.95±0.02 | 8.82±0.8 |
|  |  | 90-100 | 92.3±1.8 | 195.0±2.9 | 712.7±1.1 | 0.93 ± 0.03 | 7.42±0.4 |
| Vinasse application  (V) | | 0-10 | 297.0±1.2 | 88.3±0.4 | 614.7±1.1 | 1.10±0.00 | 21.00±0.2 |
|  |  | 10-20 | 278.0±3.3 | 96.3±1.8 | 625.7±2.3 | 1.18±0.00 | 18.24±0.2 |
|  |  | 20-30 | 294.3±3.0 | 86.3±2.7 | 619.3±4.3 | 1.22±0.00 | 15.11±0.1 |
|  |  | 30-40 | 191.3±1.7 | 117.0±1.2 | 691.7±1.5 | 1.15±0.03 | 13.28±0.9 |
|  |  | 40-50 | 177.3±0.4 | 125.7±2.2 | 697.0±2.6 | 1.00±0.01 | 9.13±0.5 |
|  |  | 50-60 | 167.0±1.9 | 95.3±1.5 | 737.7±0.4 | 0.90±0.03 | 10.60±0.9 |
|  |  | 60-70 | 176.0±2.6 | 163.0±4.3 | 661.0±4.4 | 0.95±0.02 | 8.13±0.3 |
|  |  | 70-80 | 163.0±3.1 | 128.3±0.4 | 708.7±3.5 | 0.97±0.02 | 9.20±0.3 |
|  |  | 80-90 | 170.0±0.7 | 134.0±2.5 | 696.0±3.1 | 0.93±0.03 | 8.78±0.6 |
|  |  | 90-100 | 165.3±0.8 | 89.7±2.2 | 745.0±1.4 | 0.95±0.01 | 9.27±0.9 |
| Burned  (B) | 0-10 | | 122.7±3.2 | 131.3±1.1 | 746.3±2.9 | 1.00±0.00 | 23.54±0.1 |
|  | 10-20 | | 119.0±1.4 | 123.3±2.3 | 757.7±3.5 | 1.02±0.00 | 22.27±0.1 |
|  | 20-30 | | 104.0±1.4 | 151.3±1.8 | 744.7±2.9 | 0.94±0.00 | 17.98±0.2 |
|  | 30-40 | | 114.7±0.4 | 146.3±2.7 | 739.0±2.6 | 0.99±0.04 | 11.22±0.4 |
|  | 40-50 | | 129.7±0.8 | 112.0±1.4 | 758.3±0.8 | 0.92±0.02 | 9.73±0.5 |
|  | 50-60 | | 122.7±0.4 | 126.3±2.2 | 751.0±1.9 | 0.91±0.03 | 8.30±0.2 |
|  | 60-70 | | 133.7±0.8 | 154.3±1.5 | 712.0±0.7 | 0.87±0.02 | 8.32±0.4 |
|  | 70-80 | | 167.0±2.6 | 96.3±2.9 | 736.7±3.6 | 0.86±0.03 | 7.70±0.3 |
|  | 80-90 | | 145.0±3.3 | 146.0±3.3 | 709.0±3.3 | 0.85±0.02 | 8.88±0.6 |
|  | 90-100 | | 131.3±1.1 | 125.3±1.1 | 743.3±0.4 | 0.82±0.03 | 7.67±0.2 |
| Unburned  (UB) | 0-10 | | 143.3±2.1 | 115.3±2.9 | 741.3±3.3 | 0.95±0.00 | 24.18±0.1 |
|  | 10-20 | | 133.3±2.2 | 120.7±0.4 | 746.0±2.2 | 0.92±0.00 | 22.31±0.1 |
|  | 20-30 | | 123.7±1.1 | 130.0±2.6 | 746.3±3.7 | 0.94±0.00 | 20.08±0.1 |
|  | 30-40 | | 163.0±2.6 | 175.3±1.5 | 661.7±4.1 | 0.92±0.02 | 16.67±0.4 |
|  | 40-50 | | 163.3±3.5 | 126.0±1.2 | 710.7±3.2 | 0.93±0.03 | 11.00±0.2 |
|  | 50-60 | | 159.3±1.8 | 102.3±2.2 | 738.8±4.0 | 0.90±0.03 | 9.13±0.7 |
|  | 60-70 | | 167.0±0.7 | 124.3±0.8 | 708.7±1.5 | 0.88±0.03 | 7.85±0.2 |
|  | 70-80 | | 174.6±2.9 | 124.0±1.9 | 701.3±4.8 | 0.91±0.02 | 7.05±0.1 |
|  | 80-90 | | 187.3±0.4 | 130.7±1.5 | 682.0±1.2 | 0.90±0.03 | 6.87±0.2 |
|  | 90-100 | | 166.3±2.9 | 102.7±3.0 | 731.0±5.8 | 0.89±0.02 | 5.98±0.1 |

**Table S2:** Soil chemical analyses under different sugarcane management systems

| **Management practice system** | **Depth** | **pH** | **P** | **K** | **Ca** | **Mg** | **H+Al** | **SB** | **CEC** | **BS** |
| --- | --- | --- | --- | --- | --- | --- | --- | --- | --- | --- |
|  | cm |  | mg dm^-3^ | mmolc dm^-3^ | | | | | | % |
| No Vinasse application (NV) | 0-10 | 6.1 | 13 | 2.0 | 64 | 22 | 16 | 88 | 104 | 84 |
|  | 10-20 | 5.7 | 6 | 0.5 | 37 | 14 | 28 | 52 | 80 | 65 |
|  | 20-30 | 5.3 | 4 | 0.1 | 20 | 8 | 22 | 28 | 50 | 55 |
|  | 40-50 | 5.4 | 3 | 0.1 | 23 | 5 | 28 | 28 | 55 | 50 |
|  | 90-100 | 5.6 | 1 | 0.1 | 17 | 2 | 20 | 19 | 39 | 48 |
| Vinasse application (V) | 0-10 | 4.0 | 57 | 7.9 | 28 | 11 | 79 | 46 | 126 | 37 |
|  | 10-20 | 4.6 | 58 | 6.6 | 33 | 13 | 47 | 53 | 99 | 53 |
|  | 20-30 | 4.5 | 77 | 3.5 | 14 | 6 | 58 | 23 | 81 | 29 |
|  | 40-50 | 4.7 | 3 | 1.7 | 9 | 6 | 47 | 17 | 63 | 26 |
|  | 90-100 | 4.4 | 3 | 0.3 | 9 | 5 | 42 | 14 | 56 | 25 |
| Burned (B) | 0-10 | 5.7 | 7 | 1.5 | 57 | 17 | 31 | 75 | 106 | 71 |
|  | 10-20 | 5.3 | 5 | 0.6 | 37 | 15 | 28 | 52 | 80 | 65 |
|  | 20-30 | 5.4 | 3 | 0.3 | 22 | 9 | 34 | 31 | 66 | 48 |
|  | 40-50 | 5.8 | 2 | 0.1 | 18 | 6 | 20 | 24 | 44 | 54 |
|  | 90-100 | 5.9 | 1 | 0.1 | 11 | 10 | 22 | 20 | 43 | 48 |
| Unburned (UB) | 0-10 | 4.4 | 7 | 1.5 | 21 | 11 | 52 | 33 | 86 | 39 |
|  | 10-20 | 4.8 | 5 | 3.7 | 21 | 12 | 52 | 36 | 88 | 41 |
|  | 20-30 | 4.8 | 4 | 0.5 | 12 | 7 | 34 | 20 | 54 | 37 |
|  | 40-50 | 5.8 | 2 | 0.1 | 6 | 4 | 31 | 10 | 41 | 24 |
|  | 90-100 | 4.7 | 2 | 0.1 | 2 | 2 | 25 | 4 | 29 | 15 |

SB=sum of bases, CEC=cation exchange capacity, BS=base Saturation

**Table S3:** Century parameters of cultivation (cult.100 file) by Galdos et al. 2009b; 2010a.

| **Parameter** | **Description** | **Cultivation type** | | | |
| --- | --- | --- | --- | --- | --- |
|  |  | **CT** | **P** | **M** |  |
| CULTRA(1) | fraction of aboveground live transferred to standing dead | 0 | 0 | 0 |  |
| CULTRA(2) | fraction of aboveground live transferred to surface litter | 0.05 | 0.1 | 0.05 |  |
| CULTRA(3) | fraction of aboveground live transferred to the top soil layer | 0.95 | 0.9 | 0.95 |  |
| CULTRA(4) | fraction of standing dead transferred to surface litter | 0.05 | 0.1 | 0.05 |  |
| CULTRA(5) | fraction of standing dead transferred to top soil layer | 0.95 | 0.9 | 0.95 |  |
| CULTRA(6) | fraction of surface litter transferred to top soil layer | 0.95 | 0.9 | 0.95 |  |
| CULTRA(7) | fraction of roots transferred to top soil layer | 1 | 1 | 1 |  |
| CLTEFF(1) | cultivation factor for som1 decomposition;  functions as a multiplier for increased decomposition in the month of cultivation | 1.6 | 1.6 | 5.5 |  |
| CLTEFF(2) | cultivation factor for som2 decomposition;  functions as a multiplier for increased decomposition in the month of cultivation | 5 | 1.6 | 5.5 |  |
| CLTEFF(3) | cultivation factor for som3 decomposition;  functions as a multiplier for increased decomposition in the month of cultivation | 5 | 1.6 | 1 |  |
|  | cultivation factor for soil structural material decomposition;  functions as a multiplier for increased decomposition in the month of cultivation | 5 | 1.6 | 5.5 |  |

CT: conventional tillage; P: Plowing; M: Moldboard Plow

**Table S4:** Century parameters of harvest (burn and unburned), fire and crop (sugarcane) file by Galdos et al. 2009b; 2010a.

| **Parameter** | **Description** | **Harvest** | | |
| --- | --- | --- | --- | --- |
|  |  | **Burned** | **Unburned** |  |
| AGLREM | fraction of aboveground live which will not be affected by harvest operations | 0.01 | 0.01 |  |
| BGLREM | fraction of belowground live which will not be affected by harvest operations | 0 | 0 |  |
| FLGHRV | flag indicating if grain is to be harvested | 0 | 0 |  |
| RMVSTR | fraction of the aboveground residue that will be removed | 0.9 | 0.6 |  |
| REMWSD | fraction of the remaining residue that will be left standing | 0 | 0 |  |
| HIBG | fraction of roots that will be harvested | 0 | 0 |  |
|  |  | **Fire** | |  |
|  |  | **Burned** | |  |
| FLFREM | fraction of live shoots removed by a fire event | 0.33 | |  |
| FDFREM(1) | fraction of standing dead plant material removed by a fire event | 0.33 | |  |
| FDFREM(2) | fraction of surface litter removed by a fire event | 0.85 | |  |
| FRET(1) | fraction of C in the burned aboveground material removed by a fire event | 0.1 | |  |
|  |  | **Crop** | |  |
|  |  | **Sugarcane** | |  |
| PRDX(1) | potential aboveground monthly production for crops (gC/m^2^) | 600 | |  |
| PPDF(1) | optimum temperature for production for parameterization of a Poisson Density Function curve to simulate temperature effect on growth | 30 | |  |
| PPDF(2) | maximum temperature for production for parameterization of a Poisson Density Function curve to simulate temperature effect on growth | 45 | |  |
| PPDF(3) | left curve shape for parameterization of a Poisson Density Function curve to simulate temperature effect on growth | 1 | |  |
| PPDF(4) | right curve shape for parameterization of a Poisson Density Function curve to simulate temperature effect on growth | 3.5 | |  |
| BIOFLG | flag indicating whether production should be reduced by physical obstruction; = 1 production should be reduced | 0 | |  |
| BIOK5 | level of aboveground standing dead + 10% strucc(1) C at which production is reduced to half maximum due to physical obstruction by dead material (g/m^2^) | 1800 | |  |
| PLTMRF | planting month reduction factor to limit seedling growth; set to 1.0 for grass | 1 | |  |
| FULCAN | value of aglivc at full canopy cover, above which potential production is not reduced | 150 | |  |
| FRTC(1) | initial fraction of C allocated to roots; for Great Plains equation based on precipitation, set to 0 | 0.42 | |  |
| FRTC(2) | final fraction of C allocated to roots | 0.18 | |  |
| FRTC(3) | time after planting (months with soil temperature greater than rtdtmp) at which the final value is reached | 4 | |  |
| BIOMAX | biomass level (g biomass/m^2^) above which the minimum and maximum C/E ratios of new shoot increments equal pramn(*,2) and pramx(*,2) respectively | 400 | |  |
| PRAMN(3,1) | minimum C/E ratio with zero biomass | 40 | |  |
| PRAMN(3,2) | minimum C/E ratio with biomass greater than or equal to biomax | 100 | |  |
| PRAMX(3,1) | maximum C/E ratio with zero biomass | 100 | |  |
| PRAMX(3,2) | maximum C/E ratio with biomass greater than or equal to biomax | 100 | |  |
| PRBMN(3,2) | parameters for computing minimum C/N ratio for belowground matter as a linear function of annual precipitation | 160 | |  |
| PRBMX(3,2) | parameters for computing maximum C/N ratio for belowground matter as a linear function of annual precipitation | 200 | |  |
| FLIGNI(1,1) | intercept for equation to predict lignin content fraction based on annual rainfall for aboveground material | 80 | |  |
| FLIGNI(2,1) | slope for equation to predict lignin content fraction based on annual rainfall for aboveground material. For crops, set to 0. | 200 | |  |
| FLIGNI(1,2) | intercept for equation to predict lignin content fraction based on annual rainfall for belowground material | 230 | |  |
| FLIGNI(2,2) | slope for equation to predict lignin content fraction based on annual rainfall for belowground material. For crops, set to 0. | 200 | |  |
| HIMAX | harvest index maximum (fraction of aboveground live C in grain) | 260 | |  |
| HIWSF | harvest index water stress factor; = 0 no effect of water stress; = 1 no grain yield with maximum water stress | 270 | |  |
| HIMON(1) | harvest index water stress factor;= 0 no effect of water stress; 1 no grain yield with maximum water stress | 45 | |  |
| HIMON(2) | number of months prior to harvest in which to begin accumulating water stress effect on harvest index | 390 | |  |
| EFRGRN(3) | number of months prior to harvest in which to stop accumulating water stress effect on harvest index | 340 | |  |
| VLOSSP | fraction of the aboveground E which goes to grain | 0 | |  |
| FSDETH(1) | fraction of aboveground plant N which is volatilized (occurs only at harvest) | 0 | |  |
| FSDETH(2) | maximum shoot death rate at very dry soil conditions (fraction/month); for getting the monthly shoot death rate, this fraction is multiplied times a reduction factor depending on the soil water status | 0 | |  |
| FSDETH(3) | fraction of shoots which die during senescence month; must be greater than or equal to 0.4 | 60 | |  |
| FSDETH(4) | additional fraction of shoots which die when aboveground live C is greater than fsdeth(4) | 420 | |  |
| FALLRT | the level of aboveground C above which shading occurs and shoot senescence increases | 420 | |  |
| RDR | fall rate (fraction of standing dead which falls each month) maximum root death rate at very dry soil conditions (fraction/month); for getting the monthly root death rate, this | 0 | |  |
| RTDTMP | fraction is multiplied times a reduction factor depending on the soil water status | 0 | |  |
| CRPRTF(3) | physiological shutdown temperature for root death and change in shoot/root ratio | 0 | |  |
| SNFXMX(1) | fraction of E retranslocated from grass/crop leaves at death | 0.07 | |  |
| DEL13C | symbiotic N fixation maximum for grass/crop (Gn fixed/Gc new growth) | 0 | |  |
| CO2IPR(1) | delta 13C value for stable isotope labeling | 0.1 | |  |
| CO2ITR(1) | in a grass/crop system, the effect on plant production ratio of doubling the atmospheric CO_2_ concentration from 350 ppm to 700 ppm | 0 | |  |
| CO2ICE(1,2,3) | in a grass/crop system, the effect on transpiration rate of doubling the atmospheric CO_2_ concentration from 350 ppm to 700 ppm | 0.001 | |  |
| CO2IRS(1) | in a grass/crop system, the effect on C/E ratios of doubling the atmospheric CO_2_ concentration from 350 ppm to 700 ppm | 0.3 | |  |

**Table S5:** Description of vinasse parameters in Century (omad.100) by Galdos et al. 2009b; 2010a.

| **Parameter** | **Description** | **Vinasse** | |
| --- | --- | --- | --- |
|  |  | **(g m^-2^)** |  |
| ASTGC | g of C added with addition of organic matter (g/m2) | 11.56 |  |
| ASTLBL | fraction of added C which is labeled, when C is added as a result of the addition of organic matter | 0 |  |
| ASTLIG | lignin fraction content of organic matter | 0 |  |
| ASTREC(1) | C/N ratio of added organic matter | 27.52 |  |
| ASTREC(2) | C/P ratio of added organic matter | 21.41 |  |
| ASTREC (3) | C/S ratio of added organic matter | 28.9 |  |

**Table S6.** Soil mass recovery in each fraction (light fraction, LF; heavy fraction, HF; silt and clay fraction, S+C) and total mass recovery from the original 20 g under all management practices assessed (NV) No vinasse; (V) Vinasse application; (B) Burned and (UB) Unburned.

| **Reference area** | **Depth** | **LF** | **HF** | **S+C** | **Total mass recovery** |
| --- | --- | --- | --- | --- | --- |
|  | cm | **(> 53 μm)** | **(> 53 μm)** | **(< 53 μm)** |  |
|  |  | ---------------------- g dry soil^-1^ ---------------------- | | | |
| NV | 0-10 | 0.09 | 3.68 | 16.18 | 19.95 |
| NV | 0-10 | 0.07 | 1.72 | 18.16 | 19.95 |
| NV | 0-10 | 0.11 | 2.54 | 17.09 | 19.74 |
| NV | 10-20 | 0.06 | 2.79 | 17.08 | 19.93 |
| NV | 10-20 | 0.01 | 3.01 | 16.78 | 19.80 |
| NV | 10-20 | 0.02 | 5.21 | 14.09 | 19.32 |
| NV | 40-50 | 0.38 | 8.22 | 10.87 | 19.47 |
| NV | 90-100 | 0.16 | 9.66 | 9.97 | 19.79 |
| V | 0-10 | 1.04 | 4.80 | 13.33 | 19.17 |
| V | 0-10 | 0.05 | 4.65 | 15.09 | 19.79 |
| V | 0-10 | 0.11 | 8.94 | 10.89 | 19.94 |
| V | 10-20 | 0.02 | 4.02 | 15.92 | 19.96 |
| V | 10-20 | 0.02 | 4.65 | 14.80 | 19.47 |
| V | 10-20 | 0.07 | 10.24 | 9.67 | 19.98 |
| V | 40-50 | 0.54 | 11.14 | 8.03 | 19.71 |
| V | 90-100 | 0.18 | 10.09 | 9.47 | 19.74 |
| B | 0-10 | 0.20 | 5.22 | 14.50 | 19.92 |
| B | 0-10 | 0.09 | 4.59 | 14.91 | 19.59 |
| B | 0-10 | 0.14 | 7.11 | 12.27 | 19.52 |
| B | 10-20 | 0.11 | 3.24 | 16.52 | 19.87 |
| B | 10-20 | 0.08 | 4.45 | 15.41 | 19.94 |
| B | 10-20 | 0.10 | 7.49 | 12.38 | 19.97 |
| B | 40-50 | 0.01 | 10.71 | 9.02 | 19.74 |
| B | 90-100 | 0.01 | 11.54 | 8.41 | 19.96 |
| UB | 0-10 | 0.09 | 4.49 | 15.28 | 19.86 |
| UB | 0-10 | 0.14 | 4.34 | 15.39 | 19.87 |
| UB | 0-10 | 0.01 | 4.38 | 15.31 | 19.70 |
| UB | 10-20 | 0.03 | 4.86 | 15.09 | 19.98 |
| UB | 10-20 | 0.08 | 5.78 | 14.01 | 19.87 |
| UB | 10-20 | 0.01 | 2.76 | 16.89 | 19.66 |
| UB | 40-50 | 0.02 | 9.95 | 9.53 | 19.50 |
| UB | 90-100 | 0.01 | 10.82 | 9.00 | 19.83 |


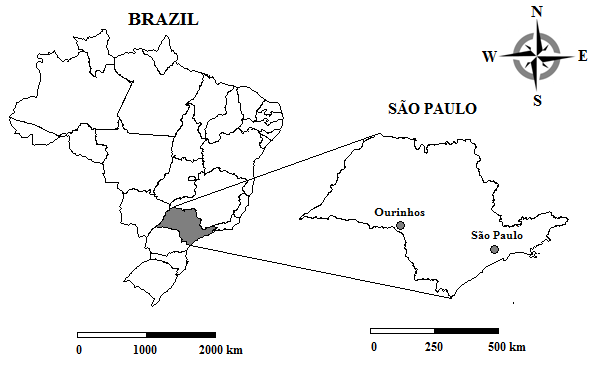


**Figure S1.** Study site location (Ourinhos) in São Paulo state, Brazil.

**Figure S2.** Average monthly rainfall and air temperature in Ourinhos, São Paulo, between 1961 to 2013. Data collected by Instituto Nacional de Metereologia (INMET).

**Figure S3.** Experimental design used for soil sampling of all sites (adapted from Mello et al. 2014


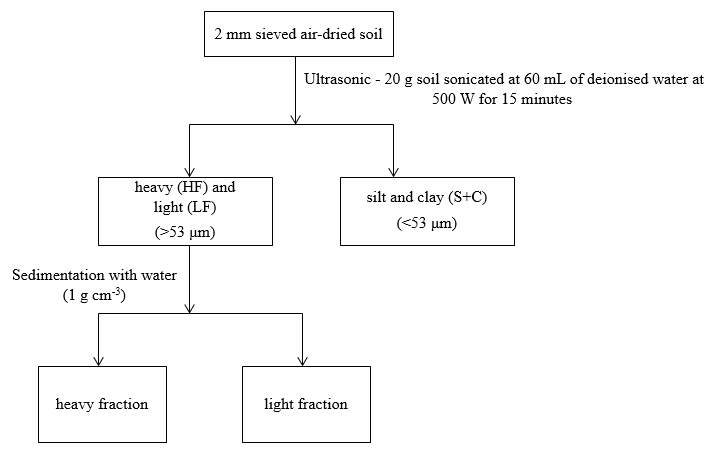


**Figure S4.** Flow diagram schematic to represent different stages of the physical and sedimentation soil fractionation (Adapted from Christensen 1985 and 1992).


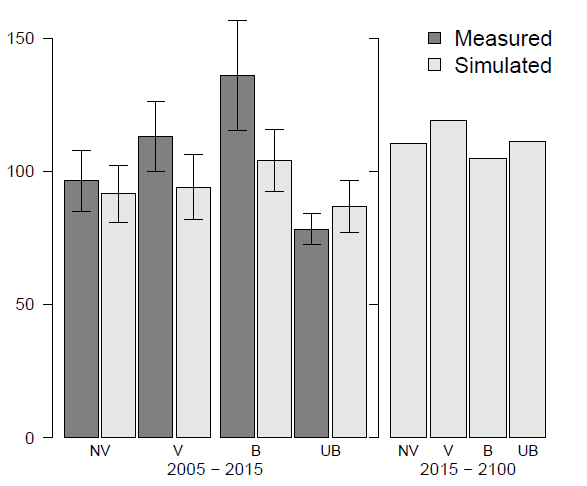


**Figure S5.** Mean measured and simulated sugarcane yields (t ha^-1^) for the harvesting events from the last 10 years (2005-2015) and for the long-term prediction (2015-2100). Vertical bars show ±1 standard error - no vinasse to vinasse (NV-to-V sites, respectively) (n=9) and burned to unburned (B-to-UB sites, respectively) (n = 6).
